# Case Report: A case series on histiocytic sarcoma – various clinical features and patient outcomes

**DOI:** 10.3389/fonc.2025.1505737

**Published:** 2025-02-24

**Authors:** Hohyung Nam, Gi-June Min, Tong Yoon Kim, Youngwoo Jeon, Seok-Goo Cho

**Affiliations:** ^1^ Department of Hematology, Seoul St. Mary’s Hematology Hospital, College of Medicine, The Catholic University of Korea, Seoul, Republic of Korea; ^2^ Department of Hematology, Yeouido St. Mary’s Hematology Hospital, College of Medicine, The Catholic University of Korea, Seoul, Republic of Korea

**Keywords:** histiocytic sarcoma, chemotherapy, salvage treatment, autologous transplantation, allogeneic transplantation

## Abstract

**Introduction:**

Histiocytic sarcoma (HS) is a rare and aggressive hematologic malignancy with a poor prognosis. HS can present with either isolated organ involvement or multi-systemic disease. This case series reports on nine patients with diverse clinical presentations and outcomes.

**Methods:**

Diagnoses of HS were confirmed using immunohistochemistry, with markers such as CD68 and lysozyme. Treatment primarily involved anthracycline-based chemotherapy with autologous hematopoietic stem cell transplantation (auto-HSCT) consolidation, and with salvage therapies for resistant or relapsed cases including allogeneic HSCT (allo-HSCT).

**Results:**

Despite intensive treatment, long-term remission was rare. Among the nine patients, three achieved complete remission but relapsed, three had stable disease, two experienced progressive disease, and one is under treatment. No patient maintained complete remission for at least three years, indicating the chemo-resistant nature of HS. Moreover, of three cases in our cohort that achieved complete remission, two declined auto-HSCT owing to the intensity of first-line chemotherapy, and one relapsed shortly after achieving remission. To overcome chemo-resistance, four patients underwent allo-HSCT, and two of them achieved long-term remission.

**Conclusion:**

These findings highlight the importance of early diagnosis and suggest potential benefits of either autologous or allogeneic transplantation, while emphasizing the need for further research on treatment protocols.

## 1 Introduction

Histiocytic sarcoma (HS) is an extremely rare hematologic malignancy originating from non-Langerhans histiocytic cells of the monocyte/macrophage lineage. These are not true sarcoma cells but are so named owing to their morphologic resemblance to mature histiocytes (1, 2). HS is diagnosed using positive immunohistochemistry for histiocytic markers, such as CD68, CD4, CD163, or lysozyme, and can present in an isolated form or be associated with other hematologic malignancies, such as non-Hodgkin lymphoma (NHL) or acute leukemia (2–4). HS has a diverse clinical presentation and can affect nearly any organ system, with the most common sites being the skin and connective tissue, followed by the lymph nodes and gastrointestinal tract (5, 6). Given the aggressive nature of HS, most patients present with multi-systemic involvement during diagnosis. HS is often life-threatening even before treatment begins and is usually treated with combined intensive chemotherapy regimens designed for aggressive NHL (4–6). However, no standard treatment for HS has been established. Furthermore, the rapid progression and poor disease prognosis may cause clinicians to miss opportunities for initiating timely and appropriate intensive treatment. Here, we report nine patients with HS with varied clinical presentations, management, and outcomes, along with a literature review.

## 2 Materials and methods

### 2.1 Clinical data collection

We retrospectively analyzed data from nine patients diagnosed with HS, with a median age of 48 years (range 26-65) and 55.6% male (n=5), in our hospital between July 2014 and May 2023. The electronic medical charts of these patients were thoroughly reviewed, and two expert clinical pathologists (each with 12 and 26 years of experience, respectively) reviewed the pathologic specimens of HS and confirmed the diagnosis using tissue morphology and immunohistochemistry. HS was positive for histiocytic markers CD68 and lysozyme and negative for dendritic cell markers CD21 and CD1a, lymphoid markers CD3 and CD20, myeloid markers, and mast cell markers (2–4). Underlying disease status, history of chemotherapies, and clinical outcomes, including relapse, disease progression, and death, were evaluated. This study was approved by the Institutional Review Board of Seoul St. Mary’s Hospital (KC24RASI0537).

### 2.2 Treatment strategy and response evaluation

A combined chemotherapy regimen designed for patients with clinically aggressive NHL was used for HS. We utilized anthracycline-based regimens CHOP (cyclophosphamide, doxorubicin, vincristine, and prednisolone), CHOEP (CHOP with etoposide), or EPOCH (etoposide, doxorubicin, and vincristine mixed into the same infusion bag and administered continuously over 24 hours for a total of 96 hours, followed by an intravenous bolus of cyclophosphamide) as the first-line treatment. When patients showed resistance to the initial chemoregimen or relapsed, ifosfamide-based (DL-ICE; dexamethasone, asparaginase, ifosfamide, carboplatin, and etoposide) or platinum-based (DHAP; dexamethasone, cytarabine, and cisplatin or ESHAP; etoposide, methylprednisolone, cytarabine, and cisplatin) regimens were used as salvage therapy. For allogeneic hematopoietic stem cell transplantation (allo-HSCT), we used a reduced-intensity conditioning regimen, as previously described (7). Response evaluation was performed via neck, chest, and abdominal/pelvic computed tomography (CT) and fluorodeoxyglucose-positron emission tomography/CT (FDG-PET/CT) after third cycles of chemotherapy or when clinically suspecting disease progression. Radiologic findings were categorized as complete remission (CR), partial remission (PR), stable disease (SD), and progressive disease (PD) according to the Lugano classification (8).

## 3 Case description

**Figure 1:**
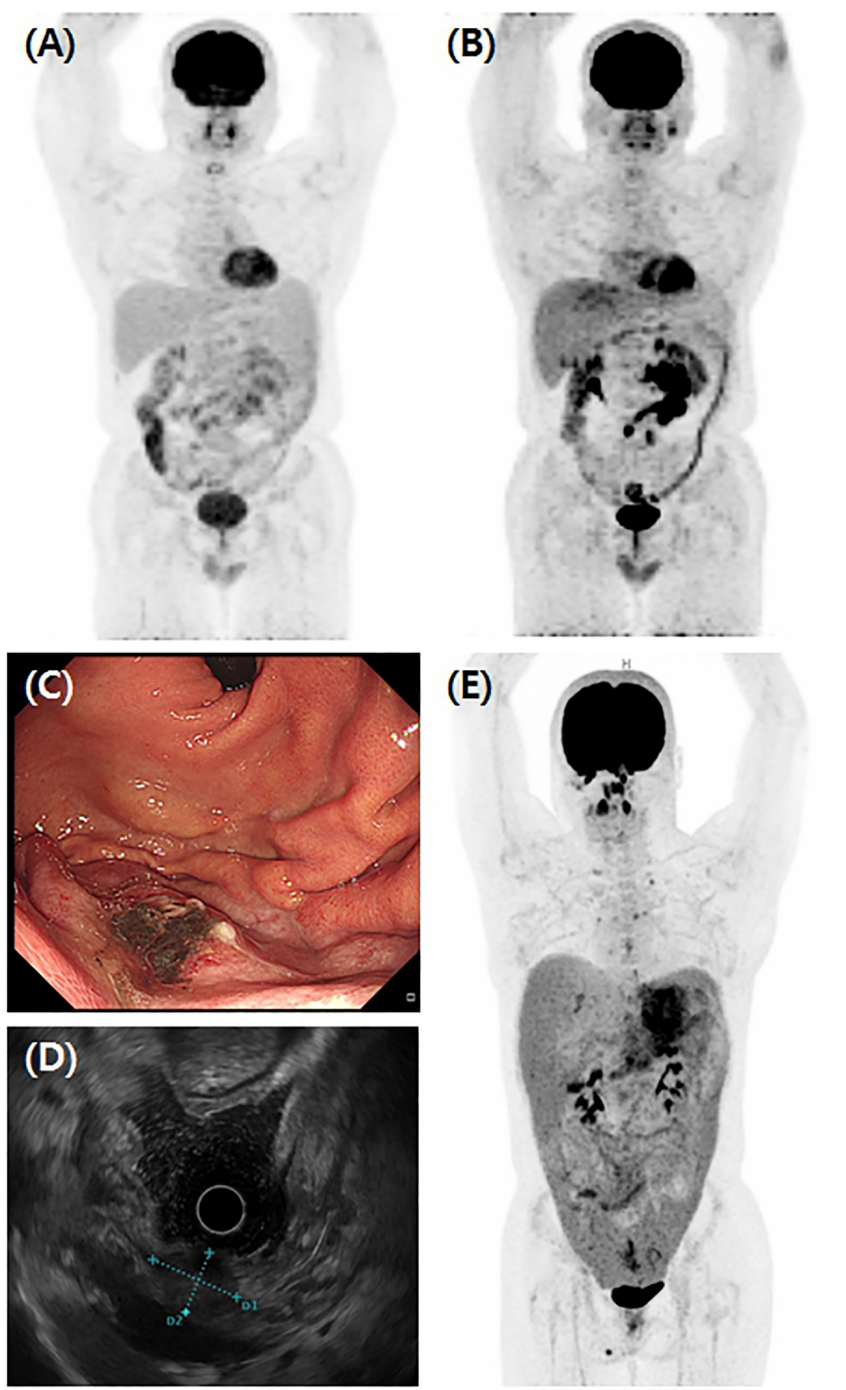
Various clinical manifestations of HS. **(A)** Initial findings of Case 1 FDG-PET/CT showed lymphoproliferative disease involving terminal ileum, ascending colon, and extensively throughout small bowel mesentery concurrent with lymph nodes in right retrocrural, aortocaval, retrocaval, and left paraaortic areas. This patient was diagnosed with follicular lymphoma and received six cycles of BR regimen. However, **(B)** follow-up FDG-PET/CT showed further increased activity of mass in mesentery and excision biopsy of mass diagnosed as HS. In Case 9, **(C)** the gastroscopic finding showed a flat elevated mass located on the high body of greater curvature, measuring about 2 cm in size. **(D)** Endoscopic ultrasound showed a 21 x 15 mm sized ovoid shape heterogeneous, hypoechoic mass with an ill-defined smooth border. **(E)** FDG-PET/CT of Case 9 showed gastric subepithelial tumor infiltrating the perigastric area and strong FDG uptakes in the peritoneum, omentum, and mesentery combined with upper abdominal, paraaortic, both iliac, and both cervical lymph nodes enlargements.

Case 1 was diagnosed with follicular lymphoma using an endoscopic biopsy of the atypical polyp-like mass in the terminal ileum and underwent six cycles of bendamustine and rituximab regimen (**Figure 1A**). However, response evaluation revealed extensive increased activity in the mesentery, suggesting high-grade lymphoma transformation and laparoscopic excision biopsy of the mesentery showed HS (**Figure 1B**). The patient underwent three cycles of EPOCH, attained SD, and switched to four cycles of ESHAP with poor response. Subsequently, Allo-HSCT from a matched sibling donor was performed as a last resort. Post-transplant, the patient developed grade 3 and 2 acute skin graft versus host disease (GVHD) and hemorrhagic cystitis (BK/JC virus negative). This process improved with hydration and steroid therapy, with no HS relapse.

Case 2 complained of constant lower abdominal pain and night sweats. The FDG-PET/CT scan showed multiple hypermetabolic lymph nodes with extensive extranodal involvement. Excision biopsy of the left supraclavicular lymph node confirmed HS diagnosis, and bone marrow (BM) biopsy revealed atypical lymphohistiocytic cells expressing CD68. Initially, the patient received EPOCH chemotherapy; however, a small bowel perforation on day 6 of the first cycle necessitated emergency resection and recovery with adequate post-operative care, granulocyte-colony stimulating factor, and antibiotics. The disease progressed despite completing three cycles of EPOCH. Subsequently, one cycle of DL-ICE was attempted but complications including persistent fever arose, during which pulmonary involvement of HS worsened. Furthermore, the treatment regimen was switched to three cycles of ESHAP, followed by allo-HSCT from a matched sibling donor while in PD. Post-transplant, the patient attained CR, experienced mild oral chronic GVHD, and has maintained remission.

Case 3 developed general weakness and visited a hospital. On admission, a peripheral blood test showed pancytopenia and an FDG-PET/CT scan revealed splenomegaly with increased FDG uptakes. A BM biopsy showed 50% cellularity, filled with 19% atypical cells showing epithelioid-to-pleomorphic morphology and positive for CD68, confirming HS diagnosis. The patient underwent three cycles of EPOCH chemotherapy; however, demonstrated SD in the BM and was transitioned to two cycles of DL-ICE. Allo-HSCT from a haploidentical donor was performed following persistent pancytopenia. Unfortunately, the patient did not recover from the pancytopenia and subsequently developed multifocal cerebral hemorrhage.

Multiple left cervical lymphadenopathies were incidentally found in Case 4, and HS was diagnosed using an excision biopsy of the left cervical lymph node in level IV. The patient completed six cycles of CHOEP and achieved CR. However, relapse occurred 33.8 months later, and PR was achieved after multiple lines of salvage chemotherapy. Allo-HSCT, gotten from a haploidentical donor was performed, resulting in approximately three years of sustained CR with grade 1 mild oral and ocular chronic GVHD. Regrettably, the patient relapsed and succumbed to the rapid progression of HS.

Case 5 had severe pelvic pain, and the FDG-PET/CT revealed a huge pelvic mass with multiple nodal and extranodal lesions; the biopsy of the pelvic mass was used to diagnose HS. The patient underwent a fourth-line chemotherapy regimen (ProMACE/CytaBOM: cyclophosphamide, doxorubicin, etoposide, and prednisone on day 1, alongside cytarabine, bleomycin, and methotrexate with leucovorin on day 8) and has maintained CR since. Transplantation was not considered in this case due to underlying psychiatric conditions.

Case 6 was presented with pancytopenia without constitutional symptoms or lymphadenopathies and was diagnosed with HS using a BM biopsy. The patient achieved CR following six cycles of CHOE; however, the patient relapsed early and presented with right-sided ptosis and diplopia. Central nervous system involvement of HS was confirmed, and CD68-positive atypical cells were identified in the cerebrospinal fluid and bone marrow biopsy. The patient received whole-brain radiotherapy (18 fractions/2520 cGy), followed by DL-ICE salvage chemotherapy. Unfortunately, the patient developed fungal pneumonia during the chemo-nadir period.

Case 7 had pelvic pain for 1 month and visited the hospital. Imaging findings showed massive lymphadenopathies and pelvic bony FDG uptakes; a core needle biopsy of left cervical lymph node level V results was used to diagnose HS. A BM biopsy also revealed HS involvement. The patient completed two cycles of CHOP; however, the response evaluation revealed PD. Subsequently, the patient received two cycles of the salvage regimen DHAP; nonetheless, PD persisted. Finally, the patient died of the disease under hospice care.

Case 8 underwent splenectomy due to splenomegaly, with suspected hematologic malignancy, and was diagnosed with HS. The patient was refractory to four cycles of CHOP and subsequently received DHAP salvage treatment. However, the disease progression continued, and the patient succumbed to progressive disease.

**Figure 2:**
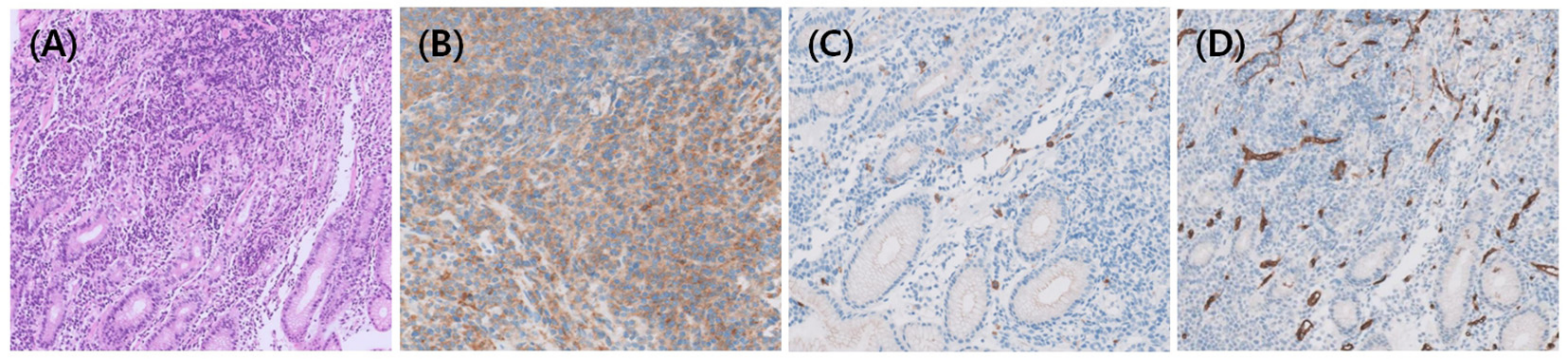
Histopathologic findings of HS. **(A)** In Case 9, Hematoxylin and eosin staining showed diffuse proliferation of neoplastic cells, which were immunohistochemically positive for **(B)** CD68 and negative for **(C)** CD117 and **(D)** CD34 to rule out GIST.

In Case 9, a 2.1 × 1.5-cm-sized ovoid-shaped gastric submucosal tumor was incidentally found at the high body of great curvature during esophago-gastro-duodenoscopy and endoscopic ultrasound (**Figures 1C, D**). The abdominal/pelvic CT findings suggested a 3.0-cm-sized subepithelial tumor infiltrating the perigastric area combined with hemoperitoneum, which raises the possibility of a ruptured gastrointestinal stromal tumor (GIST). However, an endoscopic biopsy revealed HS (**Figures 2A–D**), and FDG-PET/CT showed HS diffusely involving the skin of the right perineum, stomach, peritoneum, omentum, and mesentery lymph nodes (**Figure 1E**). Therefore, we initiated EPOCH treatment, and the patient is currently undergoing therapy. **Table 1** summarizes the clinical features of nine patients with HS.

**Table 1:** Clinical features of nine patients with histiocytic sarcoma.

No.	Age/Sex	Presenting symptoms/signs	Diagnosis site	Upper/lower diaphragm LN involvement	Extranodal sites	Initial stage	IHC results
1^*^	48/M	Painless mass	Mesentery mass	Yes/No	Mesentery and bone marrow	IV	(Positive) CD68, CD10, Bcl-6 (Negative) CD3, CD5, CD20, S-100, Pax-5, Bcl-2
2	26/M	Lower abdomen fullness for 3 months	Left cervical LN, level II	Yes/Yes	Small bowel, liver, both lungs, and sacrum	IV	(Positive) CD68 (Negative) CD3, CD20, S-100, CD1a
3	57/F	Pancytopenia	Bone marrow	No/No	Bone marrow	IV	(Positive) CD68, CD45, CD117, CD163 (Negative) CD3, CD5, CD10, CD20, CD56, CD79a
4	28/F	Painless mass	Left cervical LN, level IV	Yes/Yes	None	III	(Positive) CD68, CD4, MPO (focal) (Negative) CD3, CD10, CD20, CD45RO, S-100,CD1a
5	26/M	Both hip pain for 2 months	Huge pelvic mass	Yes/Yes	Multiple metastases in both pelvic bones, right femur, T1, T10, and L1 vertebrae with pericardial effusion	IV	(Positive) CD68, MPO (focal), S-100 (focal) (Negative) CD3, CD20, CD117, CD1a, Melan-A
6	57/M	Pancytopenia	Bone marrow	No/No	Bone marrow	IV	(Positive) CD68, CD10, CD45RB dim, Vimentin (focal) (Negative) CD30, Pax-5, CD138, ALK. CD1a,S-100
7	55/M	Both pelvic pain for 1 month	Left cervical LN, level V	Yes/Yes	Bone marrow	IV	(Positive) CD68 (Negative) CD3, CD20, CD2, CD1a, S-100, PD-1
8	65/F	Left upper abdomen fullness for 4 months; splenomegaly (long-axis diameter 19.8 cm)	Splenectomy	Yes/Yes	None	III	(Positive) CD68, S-100, Bcl-6 (Negative) CD3, CD20, CD30, Pax-5, ALK1, CD21, CD23, CD34, MPO, CD35, CD31, CD1a
9	27/F	Epigastric pain for 1 month	Gastric mass	Yes/Yes	Skin of right perineum, stomach with diffusely involving peritoneum, omentum, and mesentery	IV	(Positive) CD68, CD45RB weak (Negative) CD1a, CD20, Pax-5, MUM1, CD34, CD99, Actin,CD117, Desmin, HMB-4, S-100, Pancytokeratin

IHC, immunohistochemistry; LN, lymph node.

^*^ This patient diagnosed with grade 1 follicular lymphoma during a routine colonoscopy achieved complete remission after six cycles of BR (Bendamustine + Rituximab) chemotherapy. However, subsequent positron emission tomography-computed tomography scans revealed high-grade lymphoma transformation in the mesentery. A laparoscopic excision biopsy confirmed the presence of histiocytic sarcoma.

**Table 2** summarize the treatment course and outcomes of each enrolled patient with HS. With the first-line conventional chemotherapy, three patients achieved CR but relapsed (Cases 4–6), three had SD (Cases 1, 3, 8), two had PD (Cases 2 and 7), and one is currently under treatment (Case 9). No patient maintained long-term remission of at least 3 years and all relapsed, suggesting that HS is mostly chemo-resistant. Except for one patient currently under treatment, four of eight patients underwent allo-HSCT (Cases 1–4), and Cases 1, 2, and 4 achieved CR after transplantation. However, Case 4 relapsed and died, and Case 3 died of cerebral hemorrhage before engraftment. Two of four patients (Cases 7 and 8) treated with chemotherapy did not achieve CR and died before they could undergo transplantation. The other two (Cases 5 and 6) achieved CR but relapsed after 15.5 (Case 5) and 1.4 months (Case 6), respectively, after remission. Case 5 achieved CR and is alive following a fourth-line salvage chemotherapy regimen. In contrast, Case 6 died of infectious complications during the chemo-nadir period.

**Table 2:** Treatment modalities and outcomes of nine patients with histiocytic sarcoma.

Patient	Age/Sex	Initial treatment^†^	Initial response	From CR to relapse (months)	Salvage treatment^‡^	Salvage response	Follow-up (months)	Clinical outcomes
1	48/M	EPOCH 3 cycles	SD	N/A	ESHAP 4 cycles	SD	13.3	Allo-HSCT, alive after achieved CR
2	26/M	EPOCH 3 cycles	PD	N/A	DL-ICE 1 cycle → ESHAP 3 cycles	PD	26.3	Allo-HSCT, alive after achieved CR
3	57/F	EPOCH 3 cycles	SD	N/A	DL-ICE 2 cycles	SD	7.3	Allo-HSCT, died due to cerebral hemorrhage
4	28/F	CHOEP 6 cycles	CR→Relapse	33.8	DL-ICE 3 cycles → DHAP 3 cycles → EPOCH 2 cycles	PR	55.5	Allo-HSCT, died due to disease relapse and progression
5	26/M	EPOCH 6 cycles	CR→Relapse	15.5	DL-ICE 6 cycles → ESHAP 4 cycles → ProMACE/CytaBOM^§^ 6 cycles	CR	42.6	Alive
6	57/M	CHOEP 6 cycles	CR→Relapse	1.4	WBRT → DL-ICE 1 cycle	SD	13.2	Died due to pneumonia septic shock
7	55/M	CHOP 2 cycles	PD	N/A	DHAP 2 cycles	PD	12.7	Died due to disease progression
8	65/F	CHOP 4 cycles	SD	N/A	DHAP 1 cycle	PD	5.3	Died due to disease progression
9	27/F	EPOCH	N/A	N/A	N/A	N/A	1.6	Under treatment

Allo-HSCT, allogeneic hematopoietic stem cell transplantation; CR, complete remission; PD, progressive disease; PR, partial remission; SD, stable disease; WBRT, whole brain radiation therapy.

†We utilized anthracycline-based regimens CHOP (cyclophosphamide, doxorubicin, vincristine, and prednisolone), CHOEP (CHOP with etoposide), or EPOCH (etoposide, doxorubicin, and vincristine mixed into the same infusion bag and administered continuously over 24 h for 96 h, followed by an intravenous bolus of cyclophosphamide) as first-line treatments.

‡If patient showed resistance to the initial chemotherapy regimen or relapsed, ifosfamide (DL-ICE; dexamethasone, asparaginase, ifosfamide, carboplatin, and etoposide) or platinum-based (DHAP; dexamethasone, cytarabine, and cisplatin or ESHAP; etoposide, methylprednisolone, cytarabine, and cisplatin) regimens were used as salvage therapy.

§ProMACE/CytaBOM consisted of cyclophosphamide, doxorubicin, etoposide, and prednisone on day 1 and cytarabine, bleomycin, and methotrexate with leucovorin on day 8.

## 4 Discussion

HS is a rare, aggressive non-Langerhans histiocytic cell-derived malignancy with histologic overlap with diverse mimics, making its diagnosis challenging. Broad differential diagnosis spectrums included lymphomas, especially diffuse large B-cell lymphoma or anaplastic large cell lymphoma, poorly differentiated carcinoma, true sarcomas, dendritic cell tumors, and histiocytic disease such as Langerhans cell histiocytosis (2, 9). Therefore, clinicians’ awareness of the disease and the judicious application of immunohistochemical markers to confirm HS diagnosis are crucial. Clinicians should also be familiar with various presentations of HS, depending on which organs are involved, and most commonly, symptoms are present due to unifocal or multifocal extranodal tumors. Skin and connective tissues, lymph nodes, gastrointestinal tracts, and hematopoietic systems are well-known and frequently involving sites; however, any organ system could be involved (5, 6, 10). In our cases, two patients were diagnosed with HS using BM biopsy without any typical extranodal organ involvement (Cases 3 and 6), and another patient showed HS involving the stomach, which mimicked GIST (Case 9).

No standard treatment for HS has been established; however, surgery, radiotherapy, or systemic chemotherapy is available. Surgery followed by adjuvant radiotherapy at 45–50 Gy is a therapeutic option for unifocal HS (10). In addition, Lyizoba et al. reported a case of HS involving the tongue base that was successfully treated with radical radiotherapy (30 fractions/6000 cGy) and achieved disease-free survival exceeding 5 years (11). This case suggests that radical radiotherapy may serve as an alternative treatment option for localized HS, particularly when the potential morbidity of surgery presents significant risks or when radical surgery could severely impair the function of surrounding organs. There are no large prospective trials for multifocal HS, and the ideal chemotherapy regimen is unknown. Therefore, we used a combined chemotherapy regimen designed for patients with clinically aggressive lymphomas. We utilized anthracycline-based regimens as first-line treatments and three of eight patients achieved CR (37.5%; Cases 4, 5, and 6). However, all except one (Case 5) of the chemotherapy-based treated patients did not maintain long-term remission or even reach remission and eventually progressed. Therefore, to cure HS, four of eight patients underwent allo-HSCT (Cases 1–4), and three achieved CR.

We identified five cases of transplantation for multifocal HS in the literature; each is reported separately (**Supplementary Table S1**) (12–16). Three cases underwent autologous hematopoietic stem cell transplantation (auto-HSCT), and two cases underwent allo-HSCT. Among the three cases that underwent auto-HSCT, two procedures were performed as frontline treatments after patients achieved remission: one in CR and the other in PR (13, 14). The third case involved 3 cycles of ESHAP chemotherapy, followed by salvage auto-HSCT after relapse occurred one year following the achievement of CR with six cycles of CHOP chemotherapy (12). Two of the allo-HSCT cases with refractory multifocal HS had undergone multiple lines of treatment: one patient underwent allo-HSCT in near-CR (15), while the other was in a state of PD at the time of transplantation (16). Both patients achieved CR for 9 and 4 months, respectively, but subsequently died: one owing to pneumonia and the other following relapse and disease progression. Based on the reviewed cases, auto-HSCT consolidation may be a viable option for multifocal HS, given the chemo-resistant nature of the disease. However, this approach was not implemented in our case series. Among the three cases in our cohort that achieved CR, two patients declined auto-HSCT owing to the burdensome intensity of first-line chemotherapy, while one patient, who presented with poor performance status after achieving CR, relapsed shortly thereafter.

Kommalapati et al. reported that of 330 patients with HS, 48% were administered cyclophosphamide, doxorubicin, vincristine, and a prednisone-like regimen (chemotherapy alone, 25%; surgery + chemotherapy, 10%; radiation + chemotherapy, 9%; surgery + chemotherapy + radiation, 4%), and approximately 3% underwent allo-HSCT (6). Consequently, the median OS was 6 months, and adjuvant RT but not adjuvant chemotherapy showed OS benefits. Unfortunately, no long-term clinical outcomes of transplant patients were noted. Untreated patients had a median OS of only a few months, whereas those who received treatment had a median OS of approximately 2 years. Despite the study’s retrospective nature, small sample size, relatively short-term follow-up period, and limited data, our cases also provide valuable insights into the clinical features, and detailed clinical courses of HS in Korean patients.

In summary, owing to the highly aggressive nature of the disease and the lack of prospective trials, combined systemic chemotherapy is recommended for patients with multifocal HS. A sequence of anthracycline-based regimens followed by platinum-based or ifosfamide-based regimens, with auto-HSCT consolidation or allo-HSCT as salvage therapy, may be considered for curative potential. Further multicenter prospective studies may also help elucidate the efficacy of post-remission treatment.

## Data Availability

The datasets presented in this article are not readily available because of privacy concerns regarding patient data. Requests to access the datasets should be directed to Gi-June Min, beichest@nate.com.
