# Supplementary material for: Case Report: A case series on histiocytic sarcoma – various clinical features and patient outcomes
Source: Front Oncol. 2025 Feb 24;15:1505737. doi: 10.3389/fonc.2025.1505737 (PMC11891036; doi:10.3389/fonc.2025.1505737)
Supplement: Supplementary file 1 [file Table1.docx]

Supplementary Material

# Supplementary Tables

**Supplementary Table 1.** Five cases of transplantation in patients with multifocal histiocytic sarcoma

| **Patient** | **Age/Sex**  **Race** | **Presenting symptoms/signs** | **Diagnosis site** | **Upper / Lower diaphragm LN involvement** | **Extranodal sites** | **IHC positive** | **Treatment flow** | **Response before HSCT** | **Type of HSCT** | **Outcomes** |
| --- | --- | --- | --- | --- | --- | --- | --- | --- | --- | --- |
| 1 | 40/F  American | Recurrent abdominal pain for 3 years  Small bowel obstruction | 7 cm ileal mass | Yes/Yes | Ileal mass | (Positive) CD68,CD4,CD45,S-100, vimentin, and lysozyme  (Negative) CD2,CD3,CD5,CD7,CD8,CD20,CD21.CD23,CD30,CD43,CD1a,BLC-2,BCL-6,CK8-18, and Keratin AE 1/3 | CHOP#6→Relapsed  →ESHAP#3^‡^ | CR | ASCT  (BEAM)^§^ | CR for 24 mo |
| 2 | 38/F  Japanese | Painless subcutaneous tumor in the left calf | 3x4-cm solid mass in the subcutaneous tissue | No/Yes | Subcutaneous mass in the calf | (Positive) LCA,CD68,C100, CD163, and lysozyme  (Negative) CD1a, Langerin, CD20, CD3, CD4, CD15, CD21, EMA, and HMB45 | ^**^CHOP-AVP#3 | CR | ASCT  (BEAM) | CR for 30 mo |
| 3 | 64/F  American | Night sweats, fever, fatigue, and nonspecific flank pain | Retroperitoneal lymph node | Yes/Yes | No | (Positive) CD163, CD68, CD68R, CD31, CD45, and lysozyme  (Negative) CD1a, Langerin,S-100, CD21, CD35, CD34, vWF, paired box protein 5, CD43, CD61, CD42b, and Factor XIIIa | Thalidomide + CHOP#4→Thalidomide maintenance after ASCT | CR | ASCT  (BEAM) | CR for 6 mo |
| 4 | 33/M*  African | Throat pain  Progressing dysphagia | Laryngoscopy biopsies of irregular mass on the laryngeal surface of the epiglottis | No/No | Palatine tonsils, epiglottis, bilateral aryepiglottic folds, right Lateral hip & left upper thigh | (Positive) CD45, CD45RO, CD68, LCA, Pan-LCA, S-100 (patch), and lysozyme  (Negative) CD1a, CD21, CD30, CD3, CD20, and ALK | ^†^CHOP#3→PD→ICE→PD→CLAG-M→CR | Near CR | Allo-HSCT (MAC) | CR for 9 mo  NRM (pneumonia) |
| 5 | 51/F  American | Progressive worsening back pain  Acute onset of difficult walking | Osteoblastic lesions involving T7-8 and T12-L2 with on epidural thoracic mass | No/No | Diffuse skeletal lesions, right gluteal muscle mass, spleen, pancreatic head mass | (Positive) CD4, CD163, CD68, PD-L1 (70%)  (Negative) MPO, lysozyme, CD1a, CD15, CD21, S100, Pancytokeratin, Oscar cytokeratin, CK5/6, GATA3, TTF1, p40, PAX8, ER, SOX10, WT1 | T7-T8 decompression + palliative RT →Pembrolizumab 200 mg + CHOP#4 →PD→Pembrolizumab 200 mg + ICE #2 | PD | Allo-HSCT  (RIC) | CR for 4 mo  →Relapsed and died after 17 mo |

Allo-HSCT, allogeneic hematopoietic stem cell transplantation; ASCT, autologous stem cell transplantation; CR, complete remission; MAC, myeloablating conditioning; NRM, non-relapsed mortality; PD, progressive disease; RIC, reduced intensity conditioning; RT, radiotherapy; vWF, von-Willebrand factor

* This patient has a significant medical history of two renal transplants for glomerulonephritis and is maintained on immunosuppressive therapy with mycophenolate, tacrolimus, and prednisone.

† Except for the CHOP regimen, the number of cycles for each chemotherapy regimen was not clearly specified in the reference. CHOP consisted of cyclophosphamide, doxorubicin, vincristine, and prednisone. ICE included ifosfamide, carboplatin, and etoposide. CLAG-M consisted of cladribine, cytarabine, and mitoxantrone combined with high-dose G-CSF.

^‡^ ESHAP consists of etoposide, cisplatin, methylprednisolone, and cytarabine.

^§^ BEAM conditioning consists of carmustine, etoposide, cytarabine, and melphalan.

^**^ CHOP-AVP consists of the CHOP regimen with the addition of doxorubicin, etoposide, and prednisone.

# References

1. Abu-Sanad A, Warsi A, Michel RP, Nahal A, Popradi G, Storring JM, et al. Long-term remission after autologous stem-cell transplantation for relapsed histiocytic sarcoma. Curr Oncol (2012) 19:e289-91. doi: 10.3747/co.19.964

2. Tsujimura H, Miyaki T, Yamada S, Sugawara T, Ise M, Iwata S, et al. Successful treatment of histiocytic sarcoma with induction chemotherapy consisting of dose-escalated CHOP plus etoposide and upfront consolidation auto-transplantation. Int J Hematol (2014) 100:507-10. doi: 10.1007/s12185-014-1630-y

3. Gergis U, Dax H, Ritchie E, Marcus R, Wissa U, Orazi A. Autologous hematopoietic stem-cell transplantation in combination with thalidomide as treatment for histiocytic sarcoma: a case report and review of the literature. J Clin Oncol (2011) 29:e251-3. doi: 10.1200/JCO.2010.32.6603

4. Tomlin J, Orosco RK, Boles S, Tipps A, Wang HY, Husseman J, et al. Successful treatment of multifocal histiocytic sarcoma occurring after renal transplantation with cladribine, high-dose cytarabine, G-CSF, and mitoxantrone (CLAG-M) followed by allogeneic hematopoietic stem cell transplantation. Case Rep Hematol (2015) 2015:728260. doi: 10.1155/2015/728260

5. Huff D, Fortin Ensign S, Ryan MS, Palmer J, Munoz J. Histiocytic sarcoma treated with pembrolizumab: A case report and literature review. J Immunother Precis Oncol (2023) 6:198-202. doi: 10.36401/JIPO-23-11
